# Huge Ovarian Microcystic Stromal Tumor Presenting As Acute Abdomen in a Patient With Familial Adenomatous Polyposis: A Case Report With Magnetic Resonance Imaging Findings

**DOI:** 10.7759/cureus.72422

**Published:** 2024-10-26

**Authors:** Takuya Tsuji, Noriyoshi Oki, Tetsuo Maeda, Takahiro Watanabe, Mieko Inagaki, Shigeki Yoshida

**Affiliations:** 1 Obstetrics and Gynecology, Chibune General Hospital, Osaka, JPN; 2 Radiology, Chibune General Hospital, Osaka, JPN; 3 Pathology, Chibune General Hospital, Osaka, JPN

**Keywords:** acute abdomen, magnetic resonance imaging, microcystic stromal tumor, ovarian sex cord-stromal tumor, familial adenomatous polyposis

## Abstract

Microcystic stromal tumors (MCST) are rare sex cord-stromal tumors with distinctive microcystic features and stromal tumor immunophenotypes. Few reports have discussed MCST from the perspective of magnetic resonance imaging (MRI). In this report, we describe the MRI findings of MCST, review our case, and discuss it based on previous reports. A 24-year-old female with a history of familial adenomatous polyposis (FAP) presented to the emergency department with complaints of lower abdominal pain. A plain abdominal computed tomography scan revealed a huge ovarian tumor measuring 19 cm. MRI revealed an isointense ovarian tumor on T1-weighted images and a heterogeneous high signal intensity on T2-weighted images. Contrast-enhanced MRI demonstrated enhancement confined to the capsular structures. Based on the tumor size and MRI findings, malignancy could not be definitively excluded. However, considering the clinical presentation, a diagnosis of tumor infection was made, and an open adnexectomy was subsequently performed. Pathological examination confirmed the diagnosis of MCST, and the patient’s condition progressed without apparent recurrence one year after surgery. In previous reports, MCST has been associated with FAP, and almost all cases were benign. Patients diagnosed with FAP can reduce the risk of acute abdominal pain by using less invasive treatments, as long as they keep up with regular checkups and screenings.

## Introduction

Microcystic stromal tumor (MCST) is a rare subtype of ovarian stromal tumor first described in 2009 by Irving et al. [1]. Histologically, MCST shows microcystic, solid cellular regions and a hyalinized fibrous stroma, with immunohistological features of positive staining for vimentin, CD10, β-catenin (nuclear location), and cyclin D1 [2]. Additionally, cases of MCST in patients with familial adenomatous polyposis (FAP) with germline mutations in APC have been reported [3-5]. To our knowledge, few cases with magnetic resonance imaging (MRI) findings have been reported because of their rarity. Here, we present the radiologic findings, particularly the MRI findings, of this tumor presenting as an acute abdomen in a patient with FAP.

## Case presentation

Written informed consent was obtained from the patient for the use of patient records and any accompanying images.

**Figure 1:**
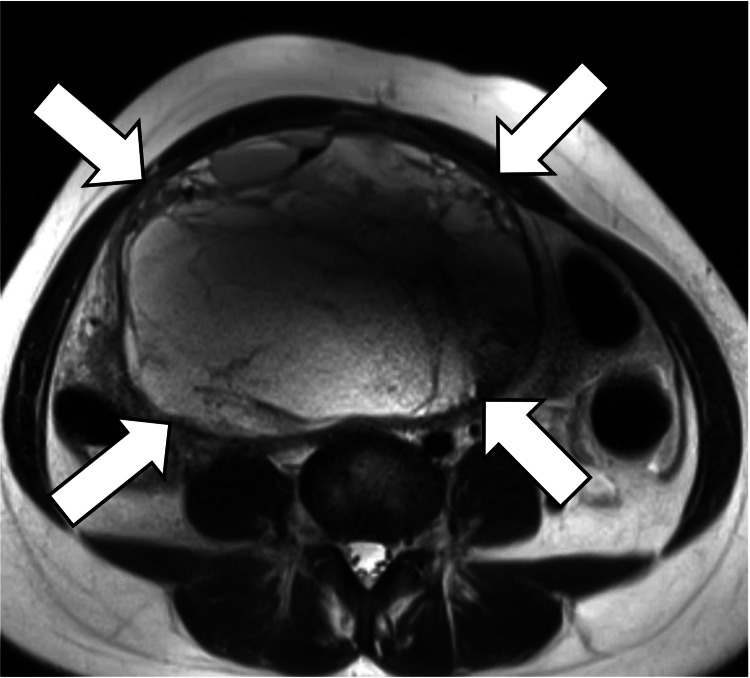
Axial, T2-weighted T2-weighted image shows a voluminous multilocular cystic mass (arrows) with several septations.

**Figure 2:**
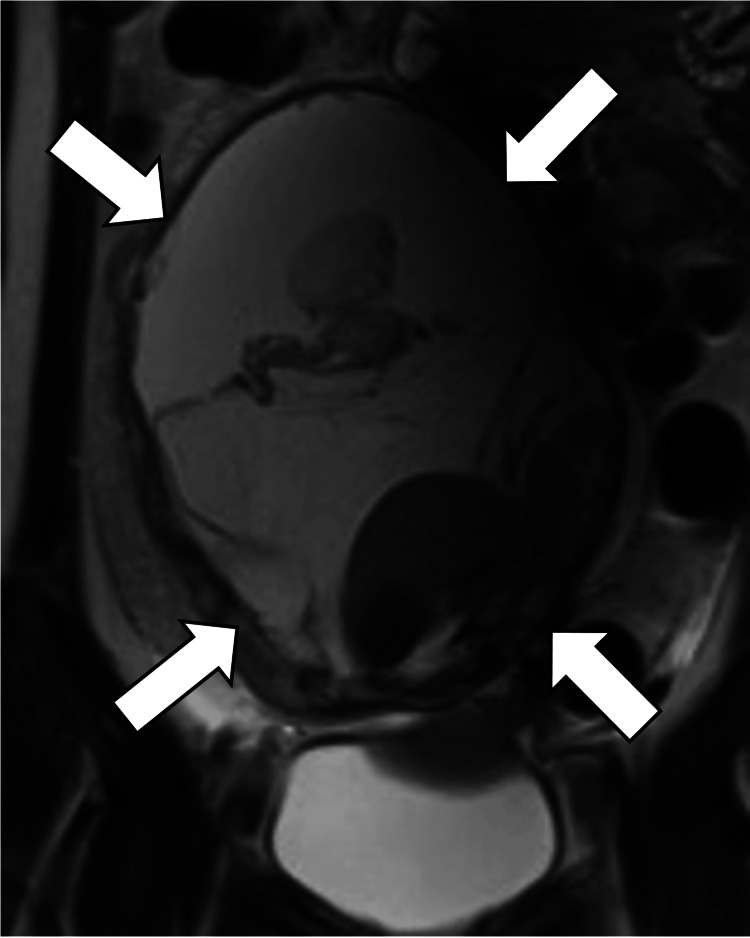
Coronal, T2-weighted T2-weighted image shows a voluminous multilocular cystic mass (arrows) with several septations.

**Figure 3:**
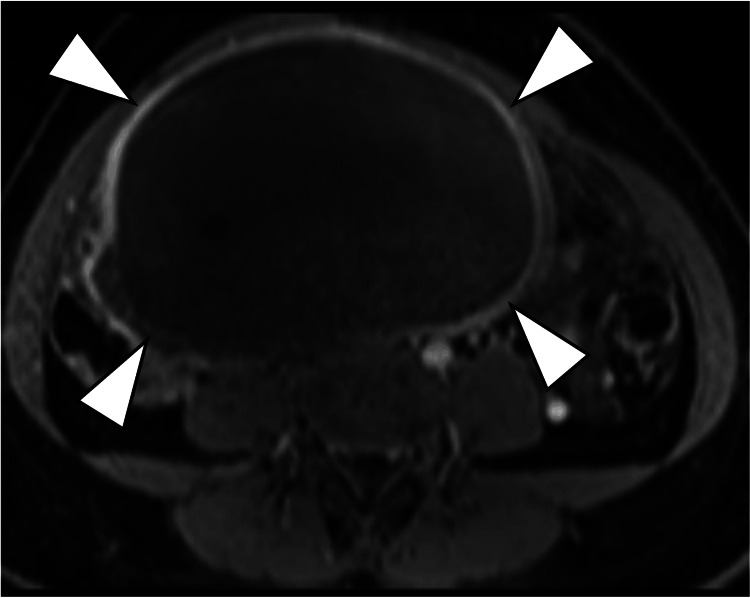
Axial, contrast-enhanced fat-suppressed T1-weighted Contrast-enhanced fat-suppressed T1-weighted image shows that only the capsular structures of the mass are enhanced (arrowheads).

**Figure 4:**
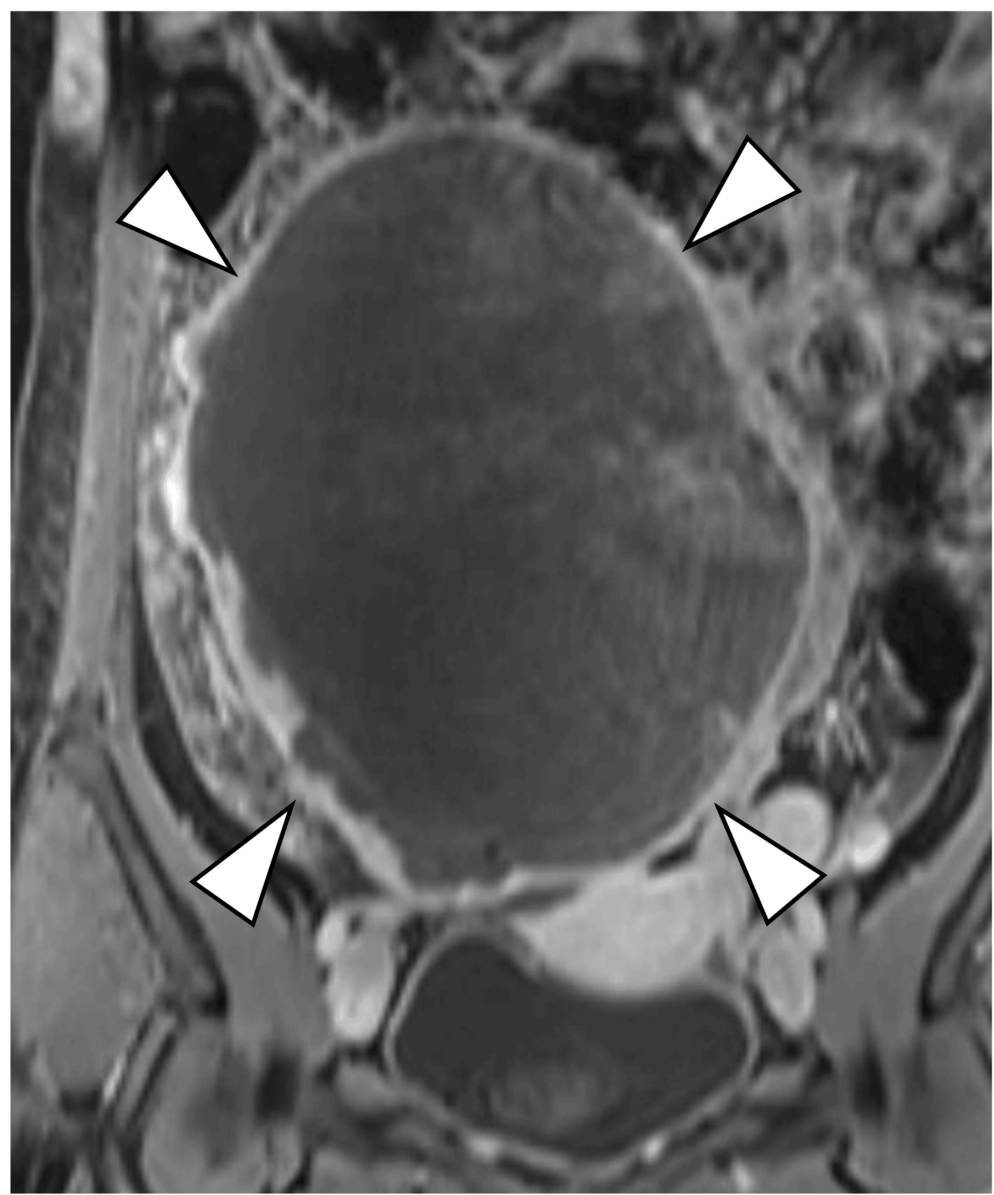
Coronal, contrast-enhanced fat-suppressed T1-weighted Contrast-enhanced fat-suppressed T1-weighted images show that only the capsular structures of the mass are enhanced (arrowheads).

A 24-year-old woman (gravida 0) presented to the emergency room with acute abdominal pain. She developed fever, nausea, and vomiting. A computed tomography (CT) scan revealed a large pelvic mass with a maximum diameter of 19 cm. On contrast-enhanced CT, the mass exhibited gradual contrast enhancement along the wall, indicating the presence of a prominent fibrous component. A lower abdomen MRI scan was performed using a 3-T Magnetom Skyra (Siemens Healthcare, Erlangen, Germany). The huge mass exhibited isointensity relative to the myometrium heterogeneous high intensity on T1- and T2-weighted images, respectively (Figures 1-2). Contrast-enhanced MRI revealed enhancement only in the capsular structure of the mass (Figures 3-4). Diffusion-weighted images were poor because of motion artifacts and were unavailable. Malignancy cannot be ruled out based on these imaging findings, including the MRI findings.

Blood tests revealed an elevated inflammatory response, with a white blood cell count of 23,800/μl and a C-reactive protein level of 33.85 mg/dl. These findings prompted the initiation of antibiotic therapy with cefmetazole and minocycline for suspected pelvic inflammatory disease (PID). However, the inflammatory response continued to escalate, and clinical symptoms such as persistent fever reaching 40.2°C, abdominal tenderness, and rebound tenderness showed no improvement. Consequently, an exploratory laparotomy was performed to control the infection, which resulted in left adnexectomy and omentectomy. The left ovary was markedly enlarged to the size of a rugby ball, with an irregular surface and partially solid areas. No evidence of torsion was noted. A dark reddish, hemorrhagic fluid with a foul odor was drained from the ovary. The uterus and right adnexa were grossly normal. The omentum was found draped over the left ovary, displaying inflammatory changes, including redness and thickening. A moderate amount of turbid, pink ascitic fluid was also observed.

**Figure 5:**
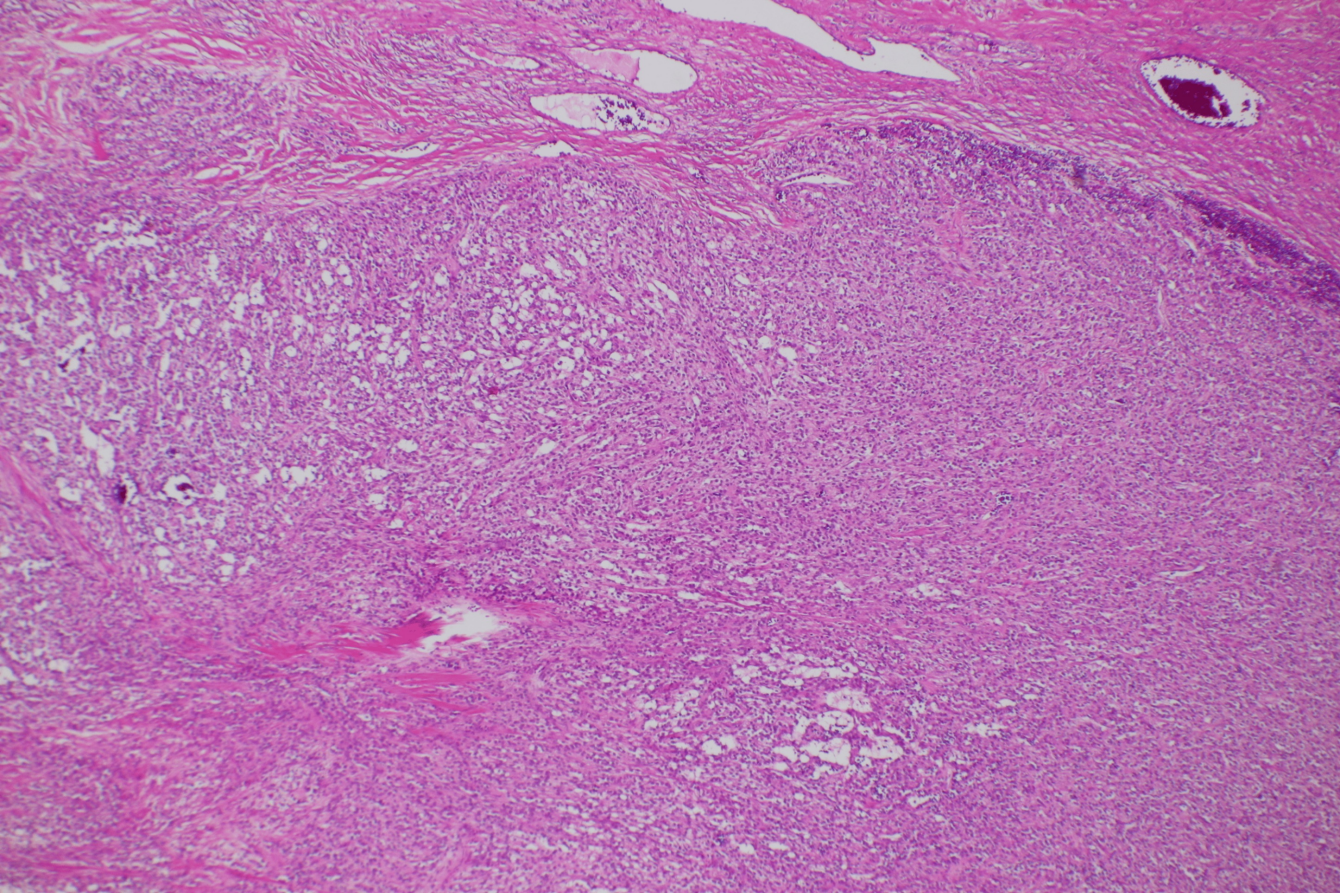
Hematoxylin and eosin staining at 40x Proliferation of substantial or microcystic cells.

**Figure 6:**
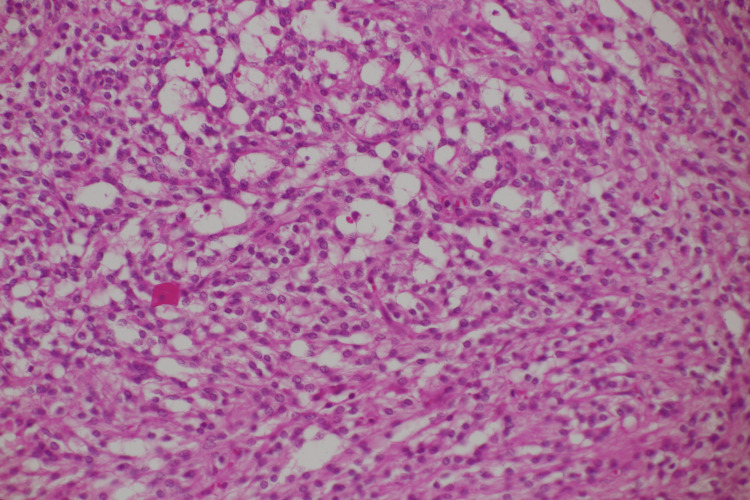
Hematoxylin and eosin staining at 200x Small, circular tumor nuclei with inconspicuous mitotic figures.

**Figure 7:**
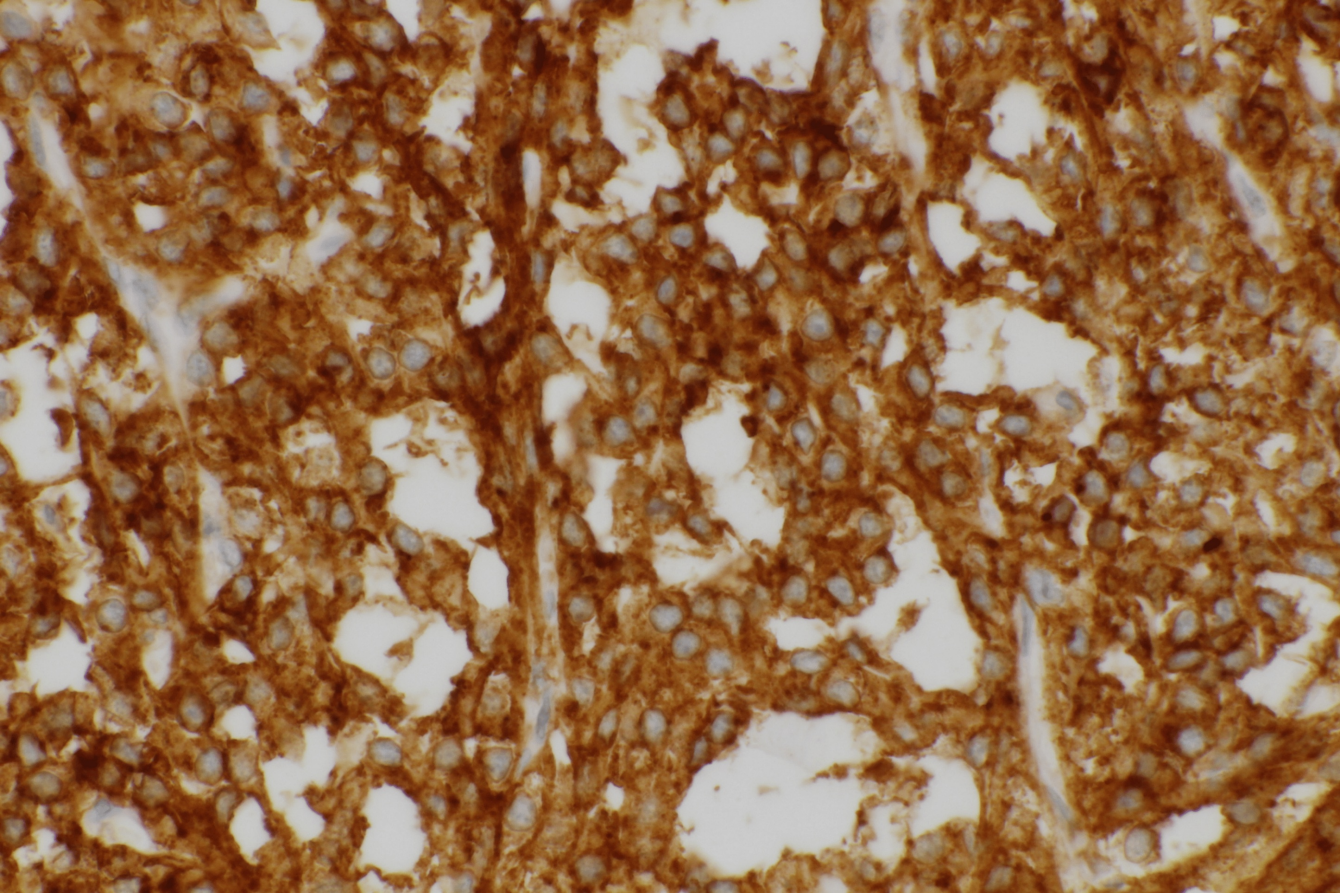
Intensely expanded immunostaining for CD10 at 400x

**Figure 8:**
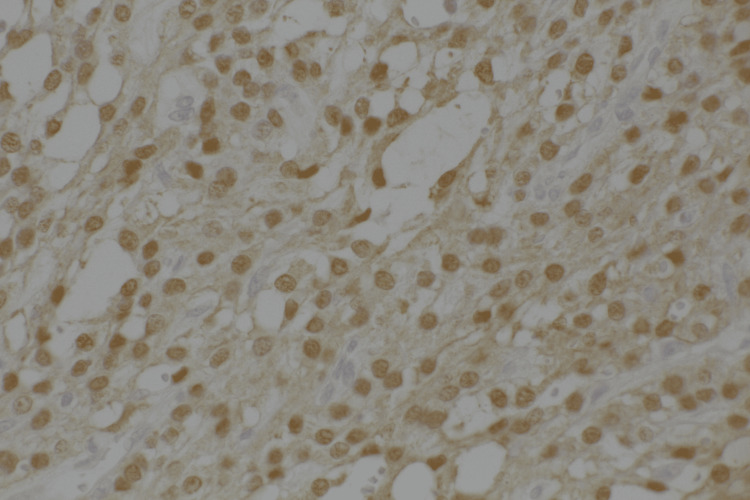
Diffuse nuclei staining with immunostaining for β-catenin at 400x

The left ovary showed a giant cystic lesion, and the cut surface revealed a grayish-white wall. Hemorrhages and hematomas were observed inside the cystic walls. Neutrophil infiltration, abscess formation, necrosis, degeneration, and hematomas were also observed. The tumor cells comprise small oval nuclei proliferating in a solid or microcystic pattern (Figure 5). Some tumor cells exhibited a signet ring cell-like morphology. Nuclear atypia was unremarkable, and mitotic figures were rare (Figure 6). Immunohistochemical examination revealed positivity for CD10, vimentin, Wilms tumor 1 (WT-1), and nuclear staining of β-catenin (Figure 7-8). The pathological examination of the omentum revealed no evidence of metastatic disease. Inhibin-α, neural cell adhesion molecule (N-CAM), AE1/AE3, estrogen receptor (ER), progesterone receptor (PgR), calretinin, alpha-smooth muscle actin (α-SMA), desmin, CD31, CD34, c-kit, SALL4, and AFP were negative. Based on these findings, the patient was diagnosed with a MCST. The histological changes mentioned above may be the result of overlapping necrosis and degeneration due to ovarian tumor stem torsion and ovarian tumor infection. Following surgery, the patient experienced a decline in inflammatory response and an improvement in general condition, leading to her discharge from the hospital. The follow-up was one year, and no tumor recurred.

## Discussion

MCST is a rare benign ovarian stromal tumor characterized by a stromal neoplasm with variable microcystic morphology, low mitotic activity, and diffuse nuclear beta-catenin and cyclin D1 immunoreactivity, while inhibin and calretinin are not expressed. This tumor may be associated with FAP [3].

Some investigators have reported cases of this condition; however, no related detailed imaging findings are available. In particular, the only case report that described MRI findings demonstrates a cystic ovarian tumor with high signal intensity on T2-weighted imaging, with a fluid-fluid level and iso-signal intensity on T1-weighted imaging [6]. In that case [6], a post-contrast study (not a dynamic study) was not performed. We present the MRI findings of this rare tumor, including dynamic studies.

On MRI, the current mass demonstrated iso intensity comparable to the skeletal muscle on T1WI (Figures 3-4) and low signal intensity mixed with a mildly high signal range on T2WI (Figures 1-2), suggesting a mixture of cystic and solid components. However, enhancement studies revealed increased enhancement of only the peripheral area (Figures 3-4). The reason why the solid components within the tumor lack enhancement is unknown, but it might be caused by ischemia due to previous ovarian torsion or venous infarction.

In previous case reports, patients were 24-69 years old [7,8] and were found to have an MCST ranging in size from 2-27 cm [1]. Tumors of all patients were discovered with abdominal distention or physical examination. Most cases are unilateral, with one occurring bilaterally [1]. Only two cases showed recurrence or metastasis [4,5], while the rest had no recurrence or metastasis. In this case, the patient was 24 years old and presented with acute abdominal pain. She had a 19 cm large multifocal tumor on the left side with smooth margins. PID, or tumor infection, can lead to adnexitis.

Laparoscopic surgery was performed in four cases. One of these was preoperatively diagnosed as a benign epithelial ovarian cyst [7], three were chocolate cysts [6,9,10], and one was scheduled for laparoscopic ovarian tumor resection; however, a laparoscopic adnexectomy was performed due to fragile tissue, suggesting malignancy and heavy bleeding [6]. Murakami et al. recommended total laparoscopic adnexectomy because of the fragility of the MCST tissue and concluded that if total laparoscopic cystectomy was performed, preparation for tumor rupture was necessary. In the present case, the preoperative diagnosis was a chocolate cyst or borderline malignant tumor 19 cm in size, and the patient underwent abdominal left adnexectomy and omentectomy. Almost all cases of the tumors have a benign course; however, MCST requires long-term observation and future research. Therefore, if MCST is suspected preoperatively and there is no obvious metastasis or peritoneal dissemination, laparoscopic surgery is a reasonable option after obtaining informed consent from the patient.

All MCSTs may harbor heterozygous mutations in the CTNNB1 gene or, less frequently, in the APC gene; in the Wnt/β-catenin pathway, inactivation of the CTNNB1 or APC gene causes accumulation of β-catenin in the cytoplasm, which translocates to the nucleus and initiates DNA transcription [11]. In the present case, the tumor was probably associated with a heterozygous mutation in APC because of the patient's history of FAP; however, we did not perform a genetic marker test. Although this patient had a family history of FAP and had previously been diagnosed with FAP at another institution, she was not followed up appropriately. No publications have described the incidence of MCST in patients with FAP. However, this patient could have been diagnosed before her ovarian tumor became massive and she experienced acute abdominal pain if she had received appropriate regular FAP screening. If the suspected preoperative diagnosis was MCST and the tumor was small, the procedure could have been performed laparoscopically.

MCST is a rare ovarian tumor, and there have been several reports of patients remaining asymptomatic until discovery. By the time of detection, the tumor often has grown large. However, in patients with FAP, which is a recognized risk factor for MCST, early detection would have been more likely. In cases like this, comprehensive screening for patients with FAP, including monitoring for ovarian tumors and other gynecological conditions, could have potentially prevented the acute abdomen caused by the giant ovarian tumor. Regular non-invasive imaging, such as transabdominal and transvaginal ultrasound, should be considered an appropriate screening approach.

## Conclusions

MCST is a relatively recently described ovarian tumor that is generally considered to follow a benign clinical course. This tumor is occasionally associated with FAP. We report a case of a patient with FAP presenting with a large ovarian tumor and acute abdomen, highlighting MRI findings that have not been so much reported in the literature. We should consider MCSTs in the differential diagnosis when patients with FAP present with ovarian tumors. Early and appropriate follow-up can facilitate less invasive treatment options, potentially preventing the progression to acute abdominal pain.
